# Supplementary material for: Dynalign II: common secondary structure prediction for RNA homologs with domain insertions
Source: Nucleic Acids Res. 2014 Nov 21;42(22):13939–48. doi: 10.1093/nar/gku1172 (PMC4267632; doi:10.1093/nar/gku1172)
Supplement: SUPPLEMENTARY DATA [file supp_gku1172_nar-02021-z-2014-File012.zip › manual/Text/TurboFold.html]

RNAstructure Command Line Help: TurboFold


|  |  |  |
| --- | --- | --- |
|  | RNAstructure Command Line Help TurboFold | - Contents - Index |
| TurboFold predicts the common structure for multiple sequences (two or more), using one of three folding modes. It has two distinct executables, a serial program called TurboFold and a parallelized program called TurboFold-smp for use in shared memory environments. USAGE 1: TurboFold <configuration file> USAGE 2: TurboFold-smp <configuration file> Required parameters: <configuration file> \| The name of a file containing required configuration data. Options which don't require added values: -h, -H, --help \| Display the usage details message. Options which require added values: NONE Configuration file format: The following is a description of valid options allowed in the configuration file. ``` ################################################################ # IMPORTANT CONFIG FILE FORMAT NOTES: # # Config file options described below are not case sensitive. # # Option lines may be specified by the option name followed by an equals sign and the option's desired value. # When specifying an option, there may be nothing else on the line. # <option> = <value> # # Specifying comment lines: # Comment lines must begin with "#" followed by a space. # There may not be more than one "#" in a comment line. # However, a comment line may be an unbroken string of "#", as in a divider between sets of options. # # Blank lines are skipped. ################################################################ ################################################################ # Input options ################################################################ # Mode specifies the resolving algorithm TurboFold uses after its initial fold. # A valid mode is required for TurboFold to run properly. # Valid modes can be one of three options: # 1. MEA (Maximum expected accuracy) # 2. ProbKnot (For pseudoknotted sequences) # 3. Threshold (Finding most probable pairs) # Modes should be specified as text strings: MEA, ProbKnot, or Threshold. # The default mode is MEA. Mode = <mode specification> # There are two separate ways to specify a group of input sequences: # 1. Place sequence file names in brackets separated by semicolons. # Note that there cannot be any spaces whatsoever between the brackets. # The list must hold "SequenceNumber" sequences. # 2. Each successive sequence "n" from 1 to SequenceNumber is specified as "Seq<n>." # Note that there cannot be any spaces in the file name. InSeq = {seq1;seq2;seq3;} Seq<n> = <seq file n> # There are two separate ways to specify a group of output CT files: # 1. Place CT file names in brackets separated by semicolons. # Note that there cannot be any spaces whatsoever between the brackets. # The list must hold "SequenceNumber" CT files. # 2. Each successive CT file "n" from 1 to SequenceNumber is specified as "CT<n>." # Note that there cannot be any spaces in the file name. OutCT = {ct1;ct2;ct3;} CT<n> = <ct file n> # SequenceNumber specifies the number of sequences given for calculation. # This is only needed if both sequences and CT files are specified individually. SequenceNumber = <number of sequences> # Note that sequence file, CT file, and sequence number options must appear in one of two valid combinations, depending if files are # specified singly or in a group. # # Singly: # Mode = <mode> # SequenceNumber = <n> # Seq1 = <seq file 1> # CT1 = <ct file n> # ... repeat single entries of Seq and CT until <n> is reached # # Groups: # Mode = <mode> # InSeq = {seq1;seq2;seq3;} # OutCT = {ct1;ct2;ct3;} # Save<1> - Save<SequenceNumber> specifies the partiton function save file names to be outputted for each successive sequence "n" # from 1 to SequenceNumber. # Note that there cannot be any spaces in the file name. # Save files are not required for any sequence. Save<n> = <save file n> # SHAPE<1> - SHAPE<SequenceNumber> specifies the SHAPE file names for each successive sequence "n" # from 1 to SequenceNumber. # SHAPE files are not required for any sequence. # Note that there cannot be any spaces in the file name. SHAPE<n> = <SHAPE file n> ################################################################ # TurboFold options ################################################################ # Note that TurboFold options affect output regardless of the mode specified. # Gamma specifies the TurboFold gamma value. # This should not be confused with MeaGamma (below). # Its default value is 0.3. Gamma = 0.3 # Iterations specifies the number of iterations TurboFold goes through. # This should not be confused with PkIterations (below). # Its default value is 3. Iterations = 3 # MaximumPairingDistance specified the maximum distance between nucleotides that can pair. # i.e. for nucleotide i to pair with j, [i - j\| < MaximumPairingDistance. # This applies to each sequence. # Its default is no limit, which is indicated by a value of zero. MaximumPairingDistance = 0 # SHAPEintercept specifies the SHAPE intercept used by TurboFold. # Note that if specified, this value is only used if one or more SHAPE files is also specified. # Its default value is -0.6 kcal/mol. SHAPEintercept = -0.6 # SHAPEslope specifies the SHAPE slope used by TurboFold. # Note that if specified, this value is only used if one or more SHAPE files is also specified. # Its default value is 1.8 kcal/mol. SHAPEslope = 1.8 # Temperature specifies the temperature at which TurboFold is run, in Kelvin. # Its default value is 310.15 K, which is 37 degrees C. Temperature = 310.15 # Processors specifies the number of processors TurboFold is run on. # Note that this flag only has an effect when TurboFold-smp, the parallel version of TurboFold, is run. # Its default value is 1. Processors = 1 ################################################################ # Maximum expected accuracy (MEA) mode options ################################################################ # Note that the following options only have an effect when MEA mode is specified. # If they are specified when TurboFold is in a different mode, they are ignored. # MaxPercent specifies the maximum percent energy difference. # Its default value is 50 (percent). MaxPercent = 50 # MaxStructures specifies the maximum number of structures to calculate. # Its default value is 1000 structures. MaxStructures = 1000 # MeaGamma specifies the MEA mode gamma value. # This should not be confused with Gamma (above). # Its default value is 1.0. MeaGamma = 1.0 # Window specifies the window size. # Its default value is 5 nucleotides. Window = 5 ################################################################ # Pseudoknot (ProbKnot) mode options ################################################################ # Note that the following options only have an effect when ProbKnot mode is specified. # If they are specified when TurboFold is in a different mode, they are ignored. # MinHelixLength is the minimum helix length allowed during folding. # Its default value is 3 nucleotides. MinHelixLength = 3 # Iterations specifies the number of iterations ProbKnot goes through. # This should not be confused with Iterations (above). # Its default value is 1. PkIterations = 1 ################################################################ # Probable Pairs (Threshold) mode options ################################################################ # Note that the following options only have an effect when Threshold mode is specified. # If they are specified when TurboFold is in a different mode, they are ignored. # Threshold specifies the probability threshold at which pairs are included in a structure. # If a threshold is explicitly specified, it should be expressed as a number >= 0.5 and <= 1.0. # Its default value is 0. # This signifies that structures should be generated at the following thresholds: # >= 0.99, >= 0.97, >= 0.95, >= 0.90, >= 0.80, >= 0.70, >= 0.60, >= 0.50 Threshold: 0 						 ``` References: 1. Harmanci, A.O., Sharma, G., and Mathews, D.H. "TurboFold: Iterative Probabilistic Estimation of Secondary Structures for Multiple RNA Sequences." *BMC Bioinformatics*, 12:108. (2011). 2. Reuter, J.S. and Mathews, D.H. "RNAstructure: software for RNA secondary structure prediction and analysis." *BMC Bioinformatics*, 11:129. (2010). | | |
| Visit The Mathews Lab RNAstructure Page for updates and latest information. | | |
